# Treatment-Decision Algorithm of Child TB: Evaluation of WHO Algorithm and Development of Indonesia Algorithm

**DOI:** 10.3390/tropicalmed10040106

**Published:** 2025-04-14

**Authors:** Rina Triasih, Finny Fitry Yani, Diah Asri Wulandari, Betty Weri Yolanda Nababan, Muhammad Buston Ardiyamustaqim, Fransiska Meyanti, Sang Ayu Kompiyang Indriyani, Tiffany Tiara Pakasi, Ery Olivianto

**Affiliations:** 1 Department of Child Health, Faculty of Medicine, Public Health and Nursing, Universitas Gadjah Mada/Dr. Sardjito Hospital, Yogyakarta 55281, Indonesia; muhammad.buston.a@mail.ugm.ac.id; 2 Department of Child Health, Faculty of Medicine, Universitas Andalas, Padang 25175, Indonesia; finny@med.unand.ac.id; 3 Department of Child Health, Faculty of Medicine, Universitas Padjajaran, Bandung 40161, Indonesia; diah.asri@unpad.ac.id; 4 Centre for Tropical Medicine, Faculty of Medicine, Public Health and Nursing, Universitas Gadjah Mada/Dr. Sardjito Hospital, Yogyakarta 55281, Indonesia; betty.nababan@penabulu-stpi.id; 5 CV Data Narya Amerta Semesta, Jakarta 13330, Indonesia; siska@dnas.co.id; 6 Department of Child Health, Faculty of Medicine, University of Mataram, Mataram 83115, Indonesia; indri_sak@unram.ac.id; 7 National Tuberculosis Program, Ministry of Health, Jakarta 12950, Indonesia; tiffany.tpakasi@kemkes.go.id; 8 Department of Child Health, Faculty of Medicine, Universitas Brawijaya, Malang 65145, Indonesia

**Keywords:** pediatric, tuberculosis, algorithm, treatment decision

## Abstract

Clinical algorithms for child tuberculosis (TB) are a valuable guide for healthcare workers to initiate treatment. We evaluated the agreement of pediatric TB diagnosis using the current Indonesia diagnostic algorithms with the 2022 WHO treatment decision algorithm and developed a new Indonesia algorithm for child TB based upon our findings and expert opinion. We conducted a retrospective study at 10 hospitals in Indonesia, involving children (0–10 years), who were evaluated for TB diagnosis in 2022. A panel of child TB experts used participants’ records to make a diagnosis using the 2022 WHO algorithm and the 2016 Indonesian algorithm. We assessed agreement between the diagnosis made by the attending doctor and those determined by the expert panel. A new Indonesia guideline was developed based on the findings and consensus of various stakeholders. Of 523 eligible children, 371 (70.9%) were diagnosed with TB by the attending doctors, 295 (56.4%) by the WHO algorithm, and 246 (47%) by the Indonesia algorithm. The Cohen’s Kappa of TB diagnosis was: attending doctor vs. WHO algorithm (0.27), attending doctor vs. Indonesia algorithm (0.45), and WHO algorithm vs. Indonesia algorithm (0.42). A review of both algorithms revealed challenges for implementation. An algorithmic approach for child TB diagnosis may not be universally applicable or implementable due to variable access to diagnostic tests and the wide variety of clinical presentations.

## 1. Introduction

It is estimated that 1.25 million children developed tuberculosis (TB) in 2022, but half of them were not diagnosed and/or not reported to the national TB programs [1]. One of the reasons for the high number of missing cases among children is the lack of confidence or competence of the healthcare worker to diagnose TB in children and the poor diagnostic performance of the currently available bacteriological tests in young children. Bacteriological confirmation of pulmonary TB (PTB) in children is challenging because of its paucibacillary nature and the difficulty in collecting sputum [2]. The yield of bacteriological examination is low, around 15% for smear microscopy and 40% for culture among children admitted to the hospital, and even lower in children attending ambulatory care, who tend to have less severe disease.

Clinicians often need to decide whether to treat for TB while awaiting the result of microbiology tests, or when the test is negative or indeterminate, or when there is no test due to inability to collect an appropriate sample or no access to the laboratory diagnostic.

Therefore, the decision is often made clinically based on a combination of symptoms, evidence of TB infection—either tuberculin skin testing (TST) or interferon gamma release assay (IGRA)—and TB-related abnormalities on the chest X-ray (CXR). The clinical diagnosis of PTB can be challenging, particularly in young children [3]. The usual symptoms of PTB (chronic cough, prolonged fever, and weight loss or failure to thrive) are common in young children living in poverty and may be caused by conditions other than TB, and so lack specificity. Tests to support a clinical diagnosis, such as CXR and tests for TB infection, are not always available in primary-level health facilities in endemic settings. Various scoring systems have been developed and applied in some countries for many decades: these are usually based on expert opinion, with adaptation based on local experience [4,5,6,7,8,9,10]. These scoring systems have not been well validated because evaluation of diagnostic accuracy is limited by the lack of a “gold” reference standard. Despite these limitations, a clinical approach that includes a scoring system has value for guiding health care workers’ decision-making for diagnosing and treating TB in children [4].

In 2022, the World Health Organization (WHO) published an example of a treatment decision algorithm for children aged < 10 years [11,12]. The treatment decision algorithm included two scoring systems: one that scored CXR findings and one that did not, for when CXR was unavailable or uninterpretable. The weighting and cut-offs for these scoring systems were developed and validated using data from over 4000 children with presumptive PTB [13]. The cut-off scores were chosen prioritizing sensitivity over specificity to minimize the number of children with TB not treated, while accepting that this would lead to overdiagnosis and the treatment of children without TB.

**Figure 1:**
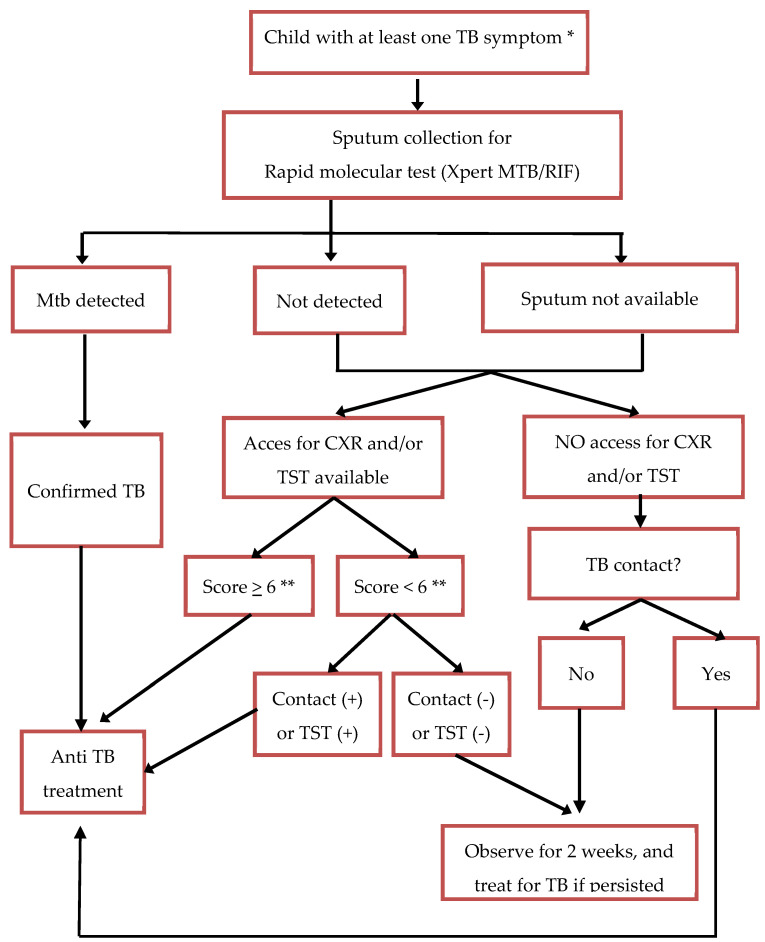
The Indonesia algorithm of child TB diagnosis 2016. * TB symptoms: chronic cough, fever more than 2 weeks, weight loss or poor weight gain. ** Use the Indonesia scoring system (Table 1).

In Indonesia, the Indonesia Pediatric Society (IPS) developed a scoring system to diagnose TB in children in 2000 (Table 1), with a total score of ≥6 leading to a diagnosis of TB disease. This scoring system had recognized limitations, such as the potential for overdiagnosis and the inability of some health facilities to perform CXR or TST [14]. Therefore, since 2016, the diagnosis of TB in children in Indonesia has been guided by an algorithm that incorporates the scoring system and provides options for those who work in limited resource settings, in which access to CXR or TST is limited (Figure 1). Following the release of the WHO treatment decision algorithm in 2022, we planned to revise the approach to diagnosis and treatment decisions for children in Indonesia. We describe the evaluation of the WHO algorithm in our setting and the process of developing the latest Indonesian algorithm.

## 2. Methods

### 2.1. Evaluation of the WHO Treatment Decision Algorithm

We conducted a retrospective study, involving 10 hospitals (eight district hospitals and two provincial hospitals) in five provinces (West Sumatra, West Java, Yogyakarta, East Java, and West Nusa Tenggara) in Indonesia. Among the 10 hospitals, all had CXR and TST, even though the availability of TST was not consistent across the hospitals. Xpert MTB/RIF was available in 9 hospitals, whereas IGRA was only available in two provincial hospitals.

The study included all children aged 0–10 years with symptoms suggestive of TB (chronic cough, fever lasting more than 2 weeks, weight loss, or poor weight gain), who were evaluated for PTB in the study hospitals in 2022. We reviewed medical records of the patients with ICD-10 code of Z03.0 (observation of suspected tuberculosis), A15 (respiratory tuberculosis, bacteriologically and histologically confirmed), A16 (respiratory tuberculosis not confirmed bacteriologically, molecularly, or histologically), or A19 (miliary tuberculosis). We excluded children whose TB symptom data were not available, and children with comorbidities other than HIV or malnutrition were excluded. When the duration of symptoms was missing, we classified the symptoms as chronic, aligning with the criteria for child TB. Children with no data on chest X-ray or TST results were not excluded and considered to reflect situations where the tests were either not performed or not accessible.

For children included in the study, we retrospectively reviewed their medical records and extracted data on symptoms, signs, results of TST or IGRA, CXR, sputum laboratory test (smear or rapid molecular diagnostic), and the final diagnosis made by the attending doctor at each hospital. We only recorded the interpretation of CXR and TST written in the medical record. A panel of pediatricians with child TB expertise and experience (RT, FFY and DAW) then assessed the data for each child using two different algorithms to make a diagnosis: the WHO algorithm published in 2022 (Algorithm A if information on the interpretation of CXR was available and Algorithm B if information on the interpretation of CXR was not available) [11,12] and the Indonesia algorithm developed in 2016 (Figure 1). Each pediatrician assessed all patients independently. In cases of discordant diagnoses, a consensus was reached through discussion involving all pediatricians in the expert panel. Each discordant case was independently re-evaluated by all experts, with each expert explaining the rationale behind their diagnosis. The final decision was determined based on agreement by at least two experts after the discussion. We then compared the number, proportion, and agreement of TB diagnosis made by the attending doctor with those made by the expert panel using the WHO algorithm and those made by the expert panel using the 2016 Indonesian algorithm. We also did a detailed review in each case.

### 2.2. Development of the New Algorithm

Based on the key findings of the study and a detailed review of some cases, the study team developed a proposed new algorithm by revising the 2016 algorithm with adaptations informed by the 2022 WHO algorithm. The principles guiding the development of the new algorithm included: (1) incorporating low-tech diagnostic steps, considering the limited availability of diagnostic tools in many settings; (2) addressing the risk of over-diagnosis; (3) emphasizing the clinical decision-making skills of clinicians, recognizing that not all patients will have findings that perfectly align with the algorithm; and (4) aligning with global algorithms while considering the local context. The proposed algorithm was then reviewed through a series of in-person and online meetings, attended by child TB experts from the IPS, representatives from the NTP, general practitioners from primary health centers, pediatricians from district hospitals, the province and district TB officers, and representatives from non-government organizations to reach consensus.

### 2.3. Data Analysis

Data were analyzed using STATA version 17 (StataCorp, College Station, TX, USA), presented as counts, mean or median, and proportion, as appropriate. Cohen’s Kappa was used to evaluate agreement in classifying TB or not TB between: the expert panel in making the diagnosis; the attending doctors and the expert panel; and the WHO algorithm and the 2016 Indonesian algorithm. Kappa scores were categorized using the following cutoffs: 0 = no agreement, 0.10–0.20 = slight, 0.21–0.40 = fair, 0.41–0.60 = moderate, 0.61–0.80 = substantial, 0.81–0.99 = near perfect, 1 = perfect). Comparison was also made on patient characteristics between those diagnosed as TB or not TB, with full agreement by all three approaches using the chi-square test for categorical variables, Wilcoxon Rank Sum test for medians, with a *p* value of less than 0.05 considered significant.

### 2.4. Ethical Clearance

We obtained ethical approval from the Medical and Health Research Ethics Committee, Faculty of Medicine, Public Health and Nursing, Universitas Gadjah Mada, Yogyakarta, Indonesia.

## 3. Results

A total of 523 eligible children were included in the study. The characteristics of the children are summarized in Table 2. More than half of the children were less than 5 years of age. Cough was the most common symptom at initial presentation. However, the duration of cough was not reported in 51 children, and the duration of fever was missing for 27 children. HIV test was not routinely performed in all children, and of those who were tested, seven children had a positive result. Most children underwent TST and CXR as part of the work-up. Microbiology tests were performed in 51.2% of the children. Of these, 13 were bacteriologically confirmed: eight on Xpert MTB/RIF, six on sputum smear, and one on both.

The proportion of children diagnosed with TB by the attending doctors was 70.9% (371/523), diagnosed by the expert panel using WHO algorithm was 56.4% (295/523) and using the Indonesia guideline was 47% (246/523) (Table 3, Table 4 and Table 5). The agreement of TB diagnosis between the attending doctor and the expert panel using the WHO algorithm was fair (Cohen’s Kappa 0.27); meanwhile, the agreement was moderate when the expert panel used the Indonesia algorithm (Cohen’s Kappa 0.45). The agreement between the WHO algorithm and the Indonesia algorithm was moderate, with Cohen’s Kappa of 0.42.

By all three approaches, there was full agreement on the diagnosis of TB in 185 children and that 99 children did not have TB. The characteristics of these children are presented and compared in Table 6. The ages of these groups were similar, but those diagnosed with TB were significantly more likely to have a known TB contact or a CXR with abnormalities suggestive of TB. Cough and poor weight gain or weight loss was also commonly reported in children without TB.

**Figure 2:**
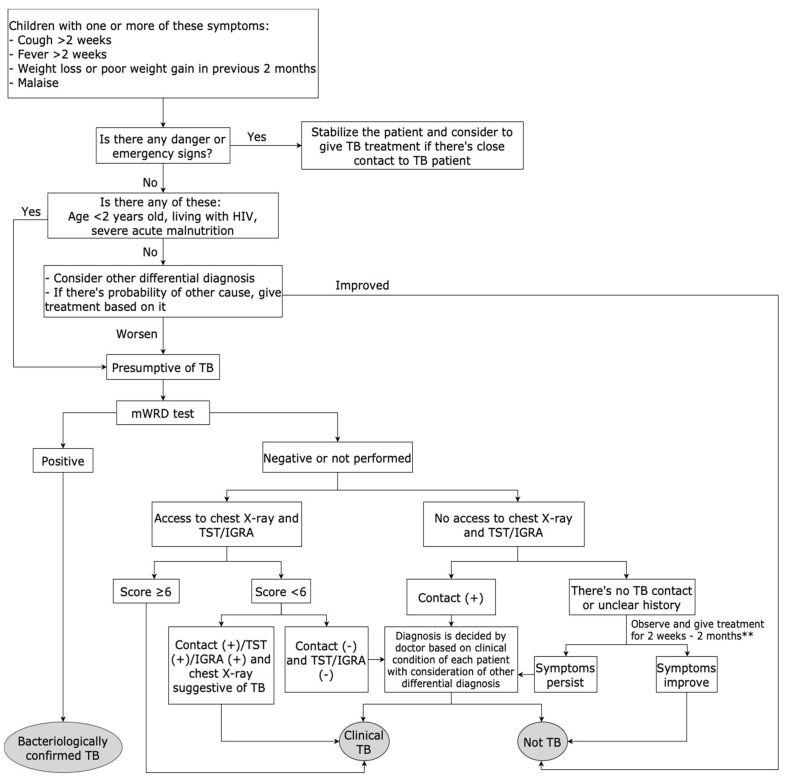
The new Indonesia algorithm to diagnose and make decisions on treating TB in children. ** non-TB treatment, based on each patient diagnosis.

The new Indonesia algorithm was developed based on some key findings in this study. First, we observed significant variability among patients in the daily practice category, which may prevent the algorithm from being followed. Therefore, we introduce a “new box” in the updated algorithm, allowing clinicians to make treatment decisions based on individual patient findings. In addition, a detailed review of the cases by the expert panel revealed a tendency toward overdiagnosis when using the 2016 Indonesia algorithm, particularly when the TB diagnosis was based solely on symptoms and evidence of infection. For example, in the case of a child with a chronic cough, normal chest X-ray, good nutritional status, and positive TST, several differential diagnoses should be considered before concluding TB. This insight led to a revision of the algorithm (Figure 2). We incorporate the identification of high-risk groups and danger signs from the WHO algorithm into the new Indonesia algorithm, as this is crucial for managing sick children. However, we did not adopt the WHO scoring system, as the expert panel’s evaluation identified a tendency of overdiagnosis. In algorithm A (when CXR is available), a total score of >10 is the threshold for initiating treatment. This means that a child with CXR indicating enlarged hilar lymph nodes (score of 17) would be treated for TB, regardless of the presence of TB infection. For example, a child with poor weight gain but no other symptoms, yet with enlarged hilar lymph nodes on the CXR, would have a total score of 20. In the absence of evidence of TB infection or close contact with a TB patient, it would be more appropriate to explore other possible causes of weight loss and treat them accordingly, rather than immediately starting a 6-month course of anti-TB treatment. In this study, of 156 children with hilar lymph nodes and enlargement on the CXR, only 95 were diagnosed as TB by the expert panel using the Indonesia algorithm.

## 4. Discussion

This study documented that doctors in hospital settings in Indonesia diagnosed TB in more children than the expert panel did. Confirming a tendency toward overdiagnosis by the attending doctor is challenging due to the lack of a reference standard, and quantifying this tendency is complicated, as the WHO and Indonesia algorithms also have a potential bias toward overdiagnosis and overtreatment. A tendency toward overdiagnosis is generally considered more acceptable than a tendency to not detect and treat children with TB, given the risk of severe disease and death, and the low risk of adverse event of anti TB treatment in this population [15]. However, best practice would indicate that unnecessary treatment should be minimized whenever possible. Therefore, a careful balance between the risks and benefit of treating or not treating TB in children should be considered before making a clinical decision. While treatment decision algorithms serve as valuable guides, the diverse characteristics of individual patients and varying access to diagnostic tools require clinicians to make a clinical decision based on each patient’s findings. Therefore, ongoing clinical training with monitoring of practice and mentorship is necessary to support healthcare workers in providing optimal decisions.

Our careful review of the WHO algorithm highlighted that a decision to treat TB disease could easily be reached by a single abnormal finding, such as hilar lymphadenopathy. Hilar lymph nodes enlargement is the most common radiological finding in the CXR of children with TB [16,17] and represents a highly specific radiological abnormality for PTB in children. However, interpreting this finding can be very challenging, especially without training [18,19,20]. The hilum, which is composed of pulmonary arteries and veins, major bronchi, and lymph nodes, is the most complex region to interpret in the chest radiogram [21,22]. Previous studies documented that inter- and intra-observer agreement in interpreting hilar lymph node enlargement on the chest radiogram was poor to moderate [23,24,25]. Insufficient experience as well as a lack of competence of doctors in reading chest radiograms in children should be taken into account. Progress is being made in computer-aided detection of common radiological patterns of TB in children, which offers promising solutions to address these challenges.

TST and IGRA are tests for TB infection and cannot distinguish between TB disease and infection. Despite the limitations, the tests can help clinicians to decide on the diagnosis of TB in children when a history of close contact with a TB patient is unknown or unclear. This was a reason for their inclusion in the Indonesia scoring system and algorithm. However, this may also lead to overdiagnosis when the diagnosis is made based on the positive results of TST/IGRA and symptoms only. On the other hand, the unavailability and lack of sustainability of these tests in health facilities can be a barrier to diagnosis if they are required as an essential component of the assessment. To address this issue, in 2016, the Indonesia algorithm was developed to include guidelines for making diagnostic decisions in the absence of TST/IGRA and CXR. This allows clinicians to start TB treatment without delay, even if these tests are not available.

The new algorithm for child TB in Indonesia adopts some of the approaches in the WHO algorithm, such as identification of danger signs and high-risk children. We did not adopt the WHO scoring system but used the scoring system developed by the IPS because of time and resource constraints to conduct a study to develop a new scoring system. This is one of the limitations of the new algorithm because the Indonesia scoring was developed based solely on literature review and expert consensus, rather than on the local data from Indonesia. Additionally, how the variables were chosen and how the weight of each variable was determined is not well-described. In the revised algorithm, we included an option allowing healthcare workers to make their own clinical decisions if the child’s clinical condition does not align with the algorithm’s guidelines. While this flexibility is intended to accommodate individual clinical judgement, it may not be suitable for all healthcare workers. A simpler and “strict” algorithm may be preferable, in particular for those with less clinical confidence and experience. There is also a risk that allowing healthcare workers discretion could lead to varying diagnostic approaches and potential overdiagnosis. As for any new tool, training and dissemination with ongoing review and mentoring of healthcare workers will be vital for uptake and maintaining the quality of care.

Ideally, external validation of the WHO algorithm should be conducted in a prospective design study and compared to the reference standard of microbiological confirmation. The main limitation of this study is its retrospective design, which relies on data from medical records that do not always provide complete and necessary information. For example, incomplete data on symptom duration should be considered limitation. This study did not compare the diagnostic approaches to a reference standard because the aim was not to evaluate the diagnostic values of the three approaches but rather to evaluate their agreement. While this may not provide robust scientific evidence, it gives a description of the diagnostic yield of both the WHO and Indonesia algorithm for TB in children. This information is important as one of the considerations for implementing the new WHO treatment decision algorithm in Indonesia. Another significant limitation was that the study did not review the chest radiograph directly; instead, it used interpretations recorded in the medical records. In addition, the retrospective nature did not allow for the control/definition of the characteristics of included children.

The use of symptoms as an entry point for screening may overlook asymptomatic cases. While asymptomatic TB cases do exist in children, the majority of childhood TB cases are identified through symptom-based screening, particularly in high-burden settings. Additionally, symptom-based algorithms remain a practical approach in resource-limited settings. To mitigate the risk of missed cases, the new algorithm emphasizes clinical judgement, allowing clinicians to evaluate children who may have suggestive risk factors despite minimal symptoms. Another limitation of the new algorithm is that the development of the algorithm was primarily based on the expert opinion. Further prospective studies are needed to evaluate the Indonesia childhood TB algorithm and to develop a new algorithm and scoring system using appropriate methods, including assessment of the diagnostic value, feasibility, and cost-effectiveness.

## 5. Conclusions

This study shows that diagnosing TB in children using an algorithm and scoring systems has limitations in interpretation and accuracy. While a simple algorithm is essential to assist healthcare workers in making clinical diagnoses and treatment decisions, especially at the peripheral levels of care, it is challenging to develop a universal algorithm applicable across all patient contexts, resources, and skill levels. A prospective evaluation of the new algorithm is needed to assess its potential for overdiagnosis and underdiagnosis, as well as the acceptability and feasibility is required. 

## Figures and Tables

**Table 1:** The 2000 Indonesia scoring system to diagnose TB in children.

	0	1	2	3
Contact	No or not clear	-	AFB ^1^ (−) or patient report	AFB ^1^ (+)
TST ^2^	Negative	-	-	Positive
Weight	-	W/A ^3^ < 80%	W/A < 60%	-
Fever	-	>2 weeks	-	-
Cough	<3 weeks	≥3 weeks	-	-
Lymph node enlargement	-	Multiple, ≥1 cm, tenderness (-)	-	-
Joint	-	Swollen	-	-
CXR ^4^	Normal	Suggestive TB ^5^	-	-

^1^ AFB: acid fast bacilli; ^2^ TST: tuberculin skin test; ^3^ W/A: weight for age; ^4^ CXR: chest X-Ray; ^5^ TB: tuberculosis.

**Table 2:** Baseline characteristics of the subjects.

Variables	N = 523 (%)
Age: median (range), years	4.1 (0.08–10.0)
Age < 5 years	313 (59.8)
Male	274 (52.4)
Known HIV-positive *	7
Initial symptom (at the time being evaluated for TB)	
Cough	382/466 (82.0)
Fever	237/420 (56.3)
Poor weight gain or weight loss	239/364 (65.7)
Reported contact with TB patient	101 (19.3)
TB work-up performed	
Tuberculin skin test	435 (83.2)
IGRA	3 (0.6)
Chest Xray	461 (88.2)
Smear microscopy	66 (12.6)
Xpert/MTB/RIF assay	202 (38.6)
Diagnosed with PTB by attending doctors	371 (70.9)
Bacteriologically confirmed TB	13/371(3.5)
Clinically diagnosed TB	358/371 (97.5)

* HIV status was unknown in majority as only recorded if positive.

**Table 3:** The agreement of TB diagnosis between the attending doctor vs. the expert panel using the WHO algorithm.

		Diagnosis by Expert Panels Using WHO Algorithm		Total
		TB	Not TB	
Diagnosis by attending doctors	TB	242	129	371
	Not TB	53	99	152
	Total	295	228	523

**Table 4:** The agreement of TB diagnosis between the attending doctor vs. the expert panel using 2016 Indonesia algorithm.

		Diagnosis by Expert Panels Using 2016 Indonesia Algorithm		Total
		TB	Not TB	
Diagnosis by attending doctors	TB	230	141	371
	Not TB	16	136	152
	Total	246	277	523

**Table 5:** The agreement of TB diagnosis made by the expert panel using the WHO algorithm vs. using 2016 Indonesia algorithm.

		Dx by Expert Panels Using 2016 Indonesia Algorithm		Total
		TB	Not TB	
Dx by expert panels using WHO algorithm	TB	188	107	295
	Not TB	58	170	228
	Total	246	277	523

**Table 6:** Characteristics of children with concordant diagnoses between all three approaches (WHO algorithm, 2016 Indonesian algorithm, and attending doctor diagnosis).

Variables	TB N = 185 (%)	Not TB N = 99 (%)	*p* Value
Age: median, (range) years	4.7 (1.8–7.6)	4.6 (2–6.7)	0.83
Age < 5 years	98 (53)	50 (50.5)	0.69
Male	100 (54.1)	58 (58.6)	0.46
HIV status known			
HIV-positive	6 (3.2%)	0	0.01
Initial symptom			
Cough	149/175(85.1)	69/88 (78.4)	0.17
Fever	87/161 (54.0)	32/81(39.5)	0.03
Poor weight gain or weight loss	107/156 (68.6)	37/56 (66.1)	0.73
Reported contact with TB patient	68 (36.8)	6 (6.1)	0.00
CXR suggestive for TB	127 (68.7)	12 (12.2)	0.00
Positive microbiology test	13 (7.0)	0	
Positive TST	162 (87.6)	0	

## Data Availability

Due to data privacy concerns, data is not made publicly available. However, reasonable data requests may be granted by contacting the corresponding author.

## Abbreviations

The following abbreviations are used in this manuscript:

TB	Tuberculosis
PTB	Pulmonary tuberculosis
WHO	World Health Organization
TST	Tuberculin skin test
IGRA	Interferon Gamma Released Assay
CXR	Chest X-Ray
IPS	Indonesia Pediatric Society
NTP	National tuberculosis program
